# Biochemical profiling of rat embryonic stem cells grown on electrospun polyester fibers using synchrotron infrared microspectroscopy

**DOI:** 10.1007/s00216-018-1049-z

**Published:** 2018-04-18

**Authors:** Ernesto Doncel-Pérez, Gary Ellis, Christophe Sandt, Peter S. Shuttleworth, Agatha Bastida, Julia Revuelta, Eduardo García-Junceda, Alfonso Fernández-Mayoralas, Leoncio Garrido

**Affiliations:** 1 Grupo de Química Neuro-Regenerativa, Hospital Nacional de Parapléjicos, Servicio de Salud de Castilla La Mancha (SESCAM), 45071 Toledo, Spain; 2 Instituto de Ciencia y Tecnología de Polímeros, Consejo Superior de Investigaciones Científicas (ICTP-CSIC), Juan de la Cierva 3, 28006 Madrid, Spain; 3 Synchrotron SOLEIL, L’Orme des Merisiers, Saint Aubin BP 48, 91192 Gif-sur-Yvette, France; 4 Instituto de Química Orgánica General, Consejo Superior de Investigaciones Científicas (IQOG-CSIC), Juan de la Cierva 3, 28006 Madrid, Spain

**Keywords:** Poly(3-hydroxybutyrate-*co*-3-hydroxyhexanoate), Electrospinning, Neural progenitor cells, FTIR spectroscopy

## Abstract

Therapeutic options for spinal cord injuries are severely limited; current treatments only offer symptomatic relief and rehabilitation focused on educating the individual on how to adapt to their new situation to make best possible use of their remaining function. Thus, new approaches are needed, and interest in the development of effective strategies to promote the repair of neural tracts in the central nervous system inspired us to prepare functional and highly anisotropic polymer scaffolds. In this work, an initial assessment of the behavior of rat neural progenitor cells (NPCs) seeded on poly(3-hydroxybutyrate-*co*-3-hydroxyhexanoate) fiber scaffolds using synchrotron-based infrared microspectroscopy (SIRMS) is described. Combined with a modified touch imprint cytology sample preparation method, this application of SIRMS enabled the biochemical profiles of NPCs on the coated polymer fibers to be determined. The results showed that changes in the lipid and amide I–II spectral regions are modulated by the type and coating of the substrate used and the culture time. SIRMS studies can provide valuable insight into the early-stage response of NPCs to the morphology and surface chemistry of a biomaterial, and could therefore be a useful tool in the preparation and optimization of cellular scaffolds.

**Electronic supplementary material:**

The online version of this article (10.1007/s00216-018-1049-z) contains supplementary material, which is available to authorized users.

**Graphical abstract:**
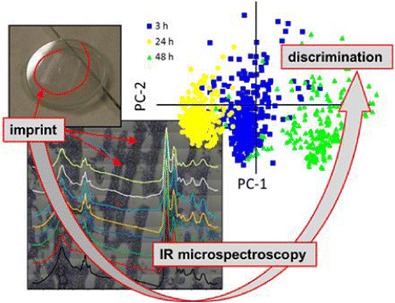
Synchrotron IR microspectroscopy can provide insight into the response of neural progenitor cells to synthetic scaffolds

## Introduction

The intrinsic characteristics of the central nervous system (CNS) are a major impediment to its spontaneous recovery in response to injury and, as a consequence, lesions cause permanent functional deficits that depend on their location and extent. In fact, complete functional repair of a spinal cord injury (SCI) cannot generally be achieved without precise and significant aid [1]. Thus, SCI is a global problem that not only affects the physical and psychological well-being of patients and their families, but also places an enormous burden on the economic resources of developed countries and increases the mortality rates in developing nations [2]. To give an example, the incidence of traumatic SCI in Western Europe was recently reported to be between 218 and 316 cases (around half of which are due to traffic accidents) per million habitants, whereas for North America (the US and Canada), the mean is over three times that [3]. In Canada alone, the annual cost of SCI in 2012 was reported to be over 2 billion €, at least 32% of which was ascribed to attendant care. In 2017, the National Spinal Cord Injury Statistical Center in the USA estimated that, depending on the severity of the SCI, the average lifetime cost of treatment and care for a person who suffers a SCI at the age of 25 was 1.4–4.3 M€ [4]. Hence, there is a great deal of interest in improving this situation.

The multifactorial nature of this type of injury severely limits therapeutic options, and the regeneration of injured axons demands a timely, structured strategy that is able to present multiple signals in a favorable environment [5, 6]. Among the diverse options that are being explored to repair neural tissue are a variety of tissue engineering approaches that are designed to facilitate the regeneration of axons using anisotropic scaffolds with biochemical cues [7–9]. These include fibers of natural and synthetic materials that mimic the spinal cord extracellular matrix and could be combined with growth factors and different types of exogenous uncommitted cells [10] or homing factors to stimulate endogenous stem cell migration towards the injury site [11].

The development of fibrous substrates resembling the extracellular matrix to support cell adhesion, differentiation, and proliferation has drawn great interest from researchers involved in tissue engineering [12–14] since the renaissance of electrospinning [15], a technique that allows the preparation of nonwoven fibers with diameters ranging from tens of nanometers to a few microns. While the tissue engineering of electrospun scaffolds has shown some limitations (i.e., it is difficult to fabricate complex three-dimensional structures and cell infiltration is poor due to small pore sizes) [16], these substrates offer the possibility of exploring cell behavior on exposure to a variety of chemical and physical cues in a reproducible manner, and they facilitate the translation of new and innovative strategies to in vivo preclinical research.

Scaffolds based on PHB [poly(3-hydroxybutyrate)] and its copolymers with other β-hydroxy acids have been employed in experimental models of CNS lesions [17, 18]. Such scaffolds offer several advantages in relation to their intended applications: they possess a reproducible and well-defined polymer microstructure, amenable to processing by a wide variety of methods, and they are both biodegradable and biocompatible. However, as is often the case for many synthetic polymers, the intrinsic surface properties of these materials do not facilitate interaction between the scaffold and the cells. Thus, the functionality of the scaffolds must be modified by the physical adsorption or covalent binding of specific chemical moieties and macromolecular fragments to its surface.

An interest in developing effective strategies to experimentally promote the repair of neural tracts in the CNS led us to prepare functional and highly anisotropic scaffolds of poly(3-hydroxybutyrate-*co*-3-hydroxyhexanoate) [P(HB-*co*-HHx)] via electrospinning. Since the physical and chemical characteristics of polymer scaffolds play an important role in the modeling of cellular behavior during growth and proliferation [19–21], the response of neural precursor cells (NPCs) to stimuli induced by P(HB-*co*-HHx) scaffolds must be characterized in order to guide and optimize approaches to SCI functional repair. In particular, studying the biochemical profile of cells and their local environment using vibrational microspectroscopy could highlight distinctive spectral markers associated with a specific cell response.

Fourier-transform infrared (FTIR) spectroscopy and (particularly) high-resolution synchrotron-based infrared microspectroscopy (SIRMS) have been successfully employed to characterize many biological tissues [22–26] and to explore several aspects of cell biology, such as cell differentiation [27–31], changes in cell physiological state [32, 33], and the effects of exogenous agents on cell biochemical profiles [34, 35]. The brightness of the synchrotron IR source provides diffraction-limited spatial resolution, allowing high-quality spectra to be obtained through small apertures that can be closely matched to the size of the cells or features of interest in the sample. The study described in the present paper applied SIRMS to search for spectral markers that allow the roles of the scaffold morphology and surface modification in the tuning and control of neural progenitor cell response during cell differentiation and subsequent proliferation to be evaluated. Spectra were obtained from NPCs cultured for up to 48 h on electrospun P(HB-*co*-HHx) scaffolds impregnated with poly-L-lysine (PLL) and laminin (L). Poly-L-lysine is a polypeptide commonly used in CNS-originated cell cultures to facilitate cell adhesion to culture plates, and laminin is a protein present in the extracellular matrix of the CNS. Samples obtained at three time points were analyzed to assess the evolution of the cell profile. We monitored variations in the extracellular matrix composition using IR spectroscopic biomarkers, particularly those associated with lipid and protein contents, during the adhesion, differentiation, and proliferation of NPCs on the scaffolds [31]. The spectroscopic findings were correlated with the results of immunochemical studies of cell morphology and membrane markers of differentiating and/or differentiated cells in culture.

## Materials and methods

### Materials

Poly(3-hydroxybutyrate-*co*-3-hydroxyhexanoate) [P(HB-*co*-HHx)] with a molar composition of 14.3% in 3-hydroxyhexanoate, molecular weight *M*_w_ = 1.9 × 10^5^ g mol^−1^ and polydispersity index *M*_w_/*M*_n_ = 1.47, was supplied by Professor Guo-Qiang Chen (Tsinghua University, China). Prior to use, the copolymer was washed with ethanol under constant stirring overnight, filtered, and vacuum dried to a constant weight. Dichloromethane supplied by Carlo Erba Réactifs-SdS (Val de Reuil, France) and methanol, ethanol, and 1,1,1,3,3,3-hexafluoro-2-propanol purchased from Sigma–Aldrich (Steinheim, Germany) were used as supplied.

Neurobasal medium and B27 supplement were purchased from GIBCO (Paisley, UK); human bFGF and EGF were from Peprotech (Rocky Hill, NJ, USA); L-glutamine, L-glutamate, penicillin, streptomycin, bisBenzimide H 33258, and a fragment of laminin containing the IKVAV sequence were from Sigma–Aldrich; and fungizone was from Invitrogen (Madrid, Spain). Rabbit polyclonal anti-GFAP was provided by Acris (Herford, Germany) and monoclonal antibody for nestin was from Santa Cruz Biotechnology (Dallas, TX, USA); antibody goat anti-rabbit IgG conjugated to Alexa Fluor 488 and antibody goat anti-rabbit IgG conjugated to Alexa Fluor 594 were provided by Molecular Probes (Eugene, OR, USA).

### Preparation of P(HB-*co*-HHx) fiber scaffolds

The fiber scaffolds were prepared by electrospinning P(HB-*co*-HHx) solutions in a mixture of solvents. Briefly, 1 g of copolymer was dissolved in 3.5 mL of a mixture of dichloromethane (3.3 mL) and 1,1,1,3,3,3-hexafluoro-2-propanol (0.2 mL) at room temperature for 12 h with constant stirring.

The copolymer solutions were immediately electrospun in a home-built apparatus consisting of a high-voltage power supply (30 kV 600 W, SL Series, Spellman, Hauppauge, NY, USA), a blunt tip needle (0.584 mm i.d.) connected to the positive pole of the power supply, and a grounded 7 cm diameter rotatable drum as collector. In addition, the flow (0.2 mL/h) of the solution through the needle and into the electric field was controlled with an infusion pump (100 Series, KD Scientific, Holliston, MA, USA). Usually, the distance between the needle tip and the rotating drum was 16 cm, the rotation speed was ~1200 rpm, and the applied voltage was 11.5 kV. The temperature in the chamber during electrospinning was maintained at around 22 °C and the relative humidity varied between 20 and 24%. Fibers were collected for 3.5 h on aluminum foil affixed to the rotating drum to obtain mechanically stable scaffolds with a mean thickness of approximately 35 μm. After vacuum drying them overnight, the deposited fibers were cut into disks (∅ = 14 mm) and stored in a dry environment at 4 °C until used.

### Animal model

The experiments followed the European Parliament and Council Directive (2013/63/EU) and the Spanish regulation (RD 53/2013) on the protection of animals for experimental use. This study was approved by our institutional animal use and care committee for animal welfare (Hospital Nacional de Parapléjicos, registered as SAPA001). Wistar rat embryos (E15) were obtained from previously anesthetized pregnant rats by cesarean section. The rats were bred and maintained at the animal house of the Hospital Nacional de Parapléjicos in Toledo.

### Neurosphere culture

Striata from E15 rats were dissected and mechanically dissociated into individual cells [36]. This cell suspension was incubated in neurobasal medium and B27 supplement containing human bFGF (10 ng/mL) and EGF (20 ng/mL) as well as L-glutamine (0.5 mM), L-glutamate (25 mM), penicillin (100 U/mL), streptomycin (0.1 mg/mL), and fungizone (2.5 lg/mL). After about 7 days in this “NB27” medium, floating neurospheres were formed and collected by low-speed centrifugation, and were washed free of glutamate by suspension in PBS and further centrifugation. Neurospheres were dissociated by mild trypsinization, passed through a 25-gauge needle, and expanded every 3–4 days.

### Neural precursor cell differentiation

After four expansion passages, NPCs grown as neurospheres for cell expansion were suspended in a 1:1 mixture of NB27 and DMEM incorporating 10% bovine serum. The neurosphere suspension was plated onto a 24-well cell culture plate that contained 14 mm ∅ disks of P(HB-*co*-HHx) fiber substrates treated with 50 μg/mL poly-L-lysine and/or 20 μg/mL of a laminin fragment containing the IKVAV sequence. ZnS IR windows coated with laminin were used as a control. The cells were incubated for 3 h to allow cell attachment and 24 and 48 h for neurosphere differentiation. After treatment, the cells were fixed for immunocytochemistry and characterized by SR-FTIR microspectroscopy.

### Immunocytochemistry

The treated cells on PLL- and/or laminin-coated substrates were fixed in 2% paraformaldehyde/sucrose in PBS (12 min, 25 °C), washed with PBS, and immunostained as follows. They were incubated for 30 min, at 25 °C, in PBS containing 1% normal goat serum with 0.1% Triton X100. The cells were then incubated (16 h at 4 °C) in the same mixture containing the primary antibody. After repeated washing with PBS, the cells on the substrates were treated with the secondary antibody (45 min, 25 °C) H 33258, 10 g/mL for 10 min at 25 °C, washed three times with PBS, mounted on a new 24-well plate with glycerol/PBS (1/1), and examined on a fluorescence microscope (DMI 6000B, Leica, Wetzlar, Germany). Mouse monoclonal anti-nestin (2Q178, Santa Cruz Biotechnology) and rabbit polyclonal anti-GFAP at 1/500 dilution were used for immunocytochemistry and revealed by secondary antibodies: goat anti-rabbit IgG conjugated to Alexa Fluor 594 or goat anti-mouse IgG conjugated to Alexa Fluor 488, respectively, at a dilution of 1/1000.

### Scanning electron microscopy

The polymer scaffolds were analyzed prior to cell culture using scanning electron microscopy (SEM). Briefly, the scaffolds were coated with approx. 5 nm Au/Pd and observed on a Philips (Eindhoven, Netherlands) XL30 scanning electron microscope at ambient temperature using the parameters indicated in each micrograph.

### Preparation of imprints for FTIR microspectroscopy measurements

To acquire IR spectra of cell cultures on polymer fibers, imprints of the substrates with cells were prepared [37]. Briefly, stored fixed samples were moistened with two drops of distilled water, placed between two 1-mm-thick ZnS IR windows, and lightly compressed for 30 min to allow the transfer of cellular material from the polymer scaffold onto the window in contact with the cell-seeded surface. After this time, the press was carefully opened and the window with the imprint was exposed to air and dried overnight at room temperature (see Fig. S1 in the “Electronic supplementary material,” ESM, for the methodology).

Cell cultures on ZnS windows were observed as prepared; no imprints were made.

ZnS windows were chosen for imprinting purposes due to their wide transparency domain in the mid-infrared and their relatively good mechanical properties, and because they are biocompatible, allowing cell growth (they show a cell viability of >90%, and cell morphologies are similar to those obtained in standard polystyrene culture flasks [38]).

### Synchrotron radiation FTIR microspectroscopy (SIRMS)

Spectra of imprints and cell clusters were recorded at the biological endstation of the SMIS beamline at Synchrotron SOLEIL, employing both bending and edge radiation from a bending magnet at a constant current (top-up mode) of around 400 mA. The spectra were obtained on a Continuum XL (Nicolet (Thermo Scientific), Waltham, MA, USA) microscope equipped with a liquid-nitrogen-cooled MCT/A detector, a 32×/NA0.65 Schwarzschild objective, a motorized knife-edge aperture, and an *xyz* motorized stage, which was coupled to a Nicolet 5700 FTIR spectrometer (Thermo Fisher Scientific, Villebon-sur-Yvette, France). The microscope was operated in dual aperture mode with a 15 × 15 μm^2^ spatial aperture. A spectral resolution of 4 cm^−1^ was achieved and 128 scans were accumulated at each data point to obtain a high signal to noise ratio.

### Data analysis

The acquired spectra were first visualized using OMNIC 9.2 (Thermo Scientific, Madison, WI, USA) in order to predefine the spectral regions of interest and to eliminate spectra exhibiting strong Mie scattering effects.

The spectra of cells and imprints were analyzed by multivariate pattern recognition techniques using The Unscrambler X, version 10.3 (Camo Software AS, Oslo, Norway). Two spectral regions, 3050–2800 cm^−1^ and 1800–1400 cm^−1^, corresponding mainly to lipid and amide I–II bands, respectively, were selected. These regions were preprocessed before analysis [third-degree polynomial, 7/7 point smoothing prior to calculation of the second derivative (third-degree polynomial, 7/7 points) and unit vector normalization]. Principal component analysis (PCA) was carried out using preprocessed, mean-centered data on the two spectral regions combined, as well as on the amide I–II bands. Four to seven principal components (PCs) were calculated using the singular-value decomposition (SVD) algorithm and leverage correction. 2D score plots and 1D loading plots were examined, respectively, to identify any spectral clustering and to obtain information on the spectral bands leading to those clusters. Unsupervised cluster analysis was performed by considering the *k*-means and Euclidean distance.

## Results

### Neural precursor cell differentiation: immunostaining

**Fig. 1a–i:**
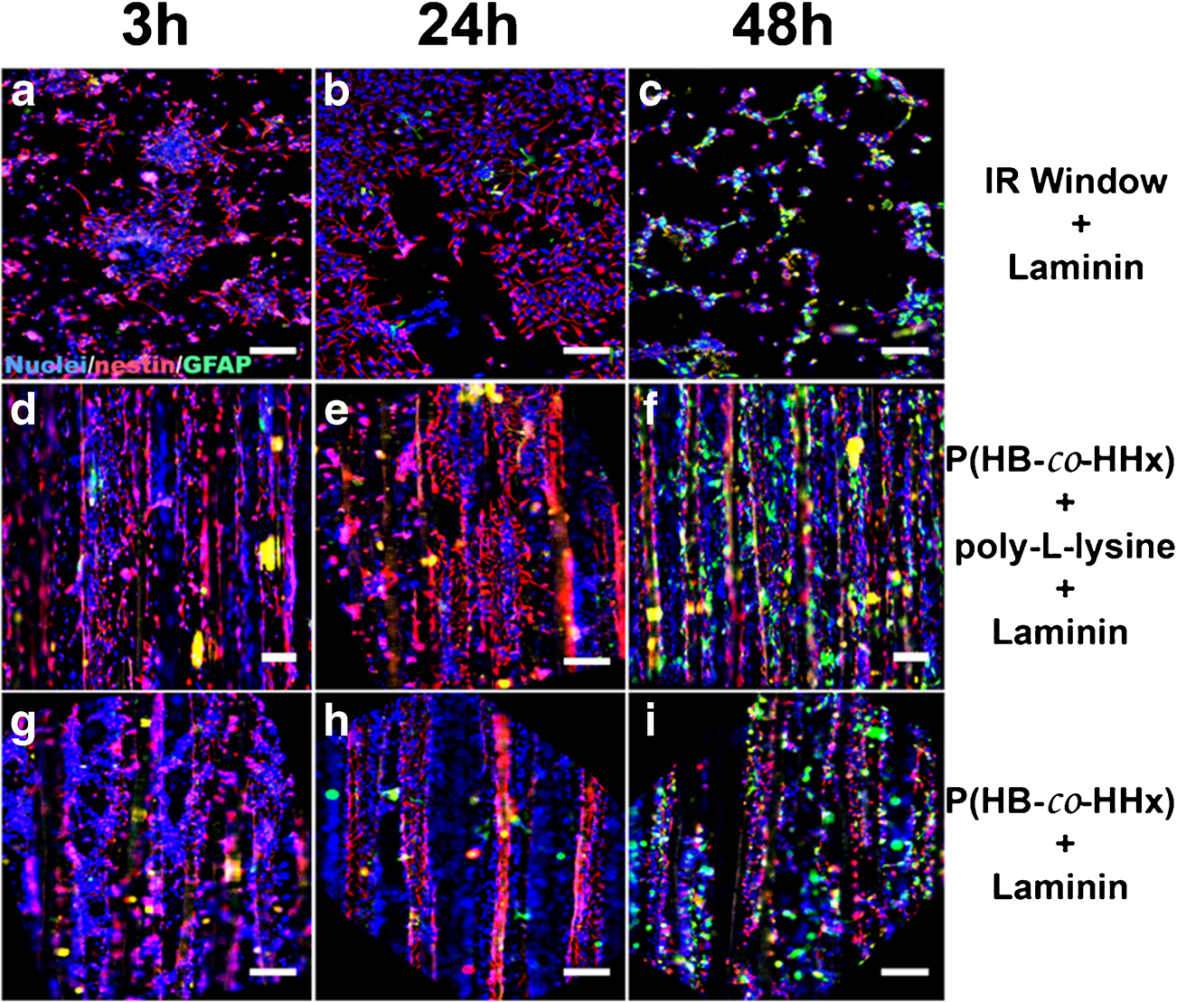
Differential nestin and GFAP expression of neural precursor cells on P(HB-*co*-HHx) substrates at specified times. The subfigures show the results of immunostaining with antibodies for nestin (*red*) and GFAP (*green*) and DNA fluorescence images of cell nuclei (*blue*). The highest number of GFAP+ cells was observed at 48 h in fibers coated with PLL/laminin (**f**), and the lowest number in laminin-coated fibers (**i**) at 48 h. *Scale bars* = 100 μm

Scaffolds of P(HB-*co*-HHx) with highly aligned fibers were prepared via electrospinning as shown in Fig. S2 (see the ESM). After coating with either laminin (L) or poly-L-lysine-laminin (PLL/L), these scaffolds were used as cell supports in cultures of E15 rat neurospheres that were isolated and cultured for up to 48 h, as described in the “Methods” section. In addition to the cultures on P(HB-*co*-HHx) fibers, zinc sulfide (ZnS) IR windows coated with laminin were used as a positive control for NPC differentiation. The cells were incubated in the presence of serum to favor cell adhesion and cell differentiation, and were fixed for immunocytochemistry after the specified time had elapsed. The results are illustrated in Fig. 1. At 3 h, aggregated NPCs that were effectively attached to substrates were observed, with a low expression of GFAP (green signal) and a high expression of nestin (red signal; see Fig. 1a, d, g). Nestin is an intermediate filament protein that is widely considered a biomarker of neural progenitor cells [39]. Therefore, during the initial stages of NPC culture on the polymer substrates, high expression is anticipated, but this expression is expected to reduce as the culture and cell differentiation progresses. At 24 h, the NPCs had migrated and colonized the substrates as individual cells, but higher expression of nestin than GFAP was still observed (Fig. 1b, e, h). The highest number of GFAP+ cells was obtained for substrates coated with PLL/laminin, contrasting with the low GFAP signal seen for laminin-coated substrates, as demonstrated for the P(HB-*co*-HHx) substrates by Fig. 1f and i, respectively (taken at 48 h). The intracellular expression of nestin and GFAP intermediate filaments detected with the specified antibodies revealed the presence of cells with precursor and astroglial phenotypes, respectively. The cell populations had blue-stained nuclei.

### FTIR microspectroscopy

**Fig. 2:**
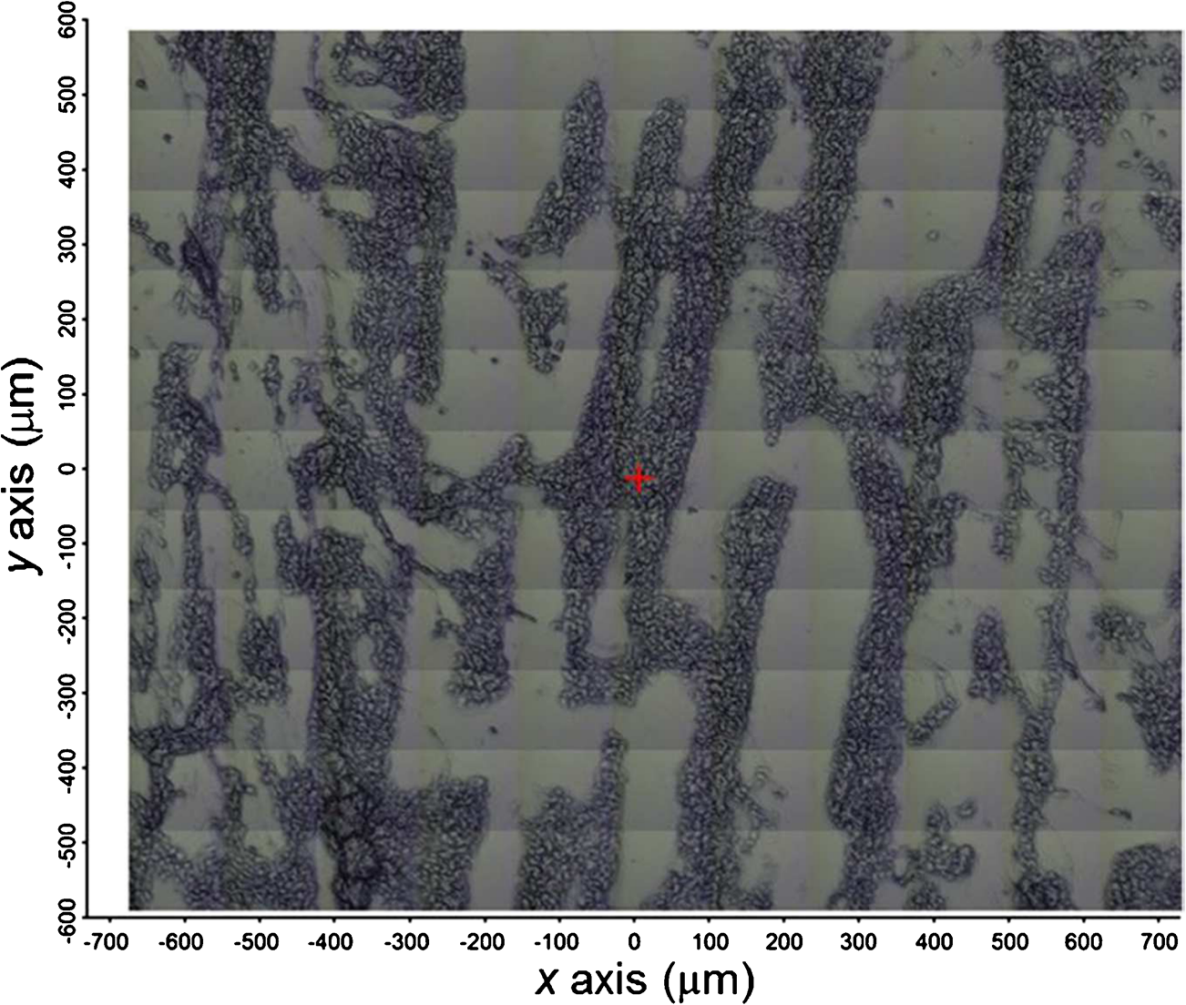
Mosaic of bright-field micrograph images (approx. 1.4 mm × 1.2 mm) corresponding to NPCs on P(HB-*co*-HHx) fibers coated with poly-L-lysine and laminin, obtained after 24 h of culture

The measured sample sets consisted of laminin-coated ZnS IR windows and P(HB-*co*-HHx) fibers coated with only laminin (L) or with both poly-L-lysine and laminin (PLL/L) for culture times of 3, 24, and 48 h. Initial attempts to measure the cells grown on the fiber scaffolds using ATR microspectroscopy through a ZnSe hemisphere [40, 41] were hampered by two fundamental problems: significant distortion in the IR spectral background was observed due to the Mie scattering effect generated by the cells due to the irregular topography of the fiber substrates, and the overwhelmingly strong IR absorption bands associated with the polymer fibers. To overcome these issues, imprints of the cells grown on the polymer scaffolds were made to transfer the cells from the polymer scaffolds to ZnS infrared transparent substrates, as described in the “Methods” section (and in Fig. S1 of the ESM), and these were subsequently measured by transmission FTIR microspectroscopy. A similar approach has been successfully employed by Das et al. [37] to study fresh tissue using FTIR. Figure 2 shows a characteristic imprint corresponding to a sample of NPCs on P(HB-*co*-HHx) fibers coated with PLL/L, which was obtained 24 h after seeding NPCs. The dark areas contain the cellular material that was pressure-transferred from the scaffold to the IR window. Although it is possible that the material was incompletely transferred from the polymer fibers to the ZnS windows, the variation in the outcome of the spectroscopic measurements for imprints of a given experimental setting was insignificant, or at least far smaller than the variations caused by changing the culture conditions.

**Fig. 3:**
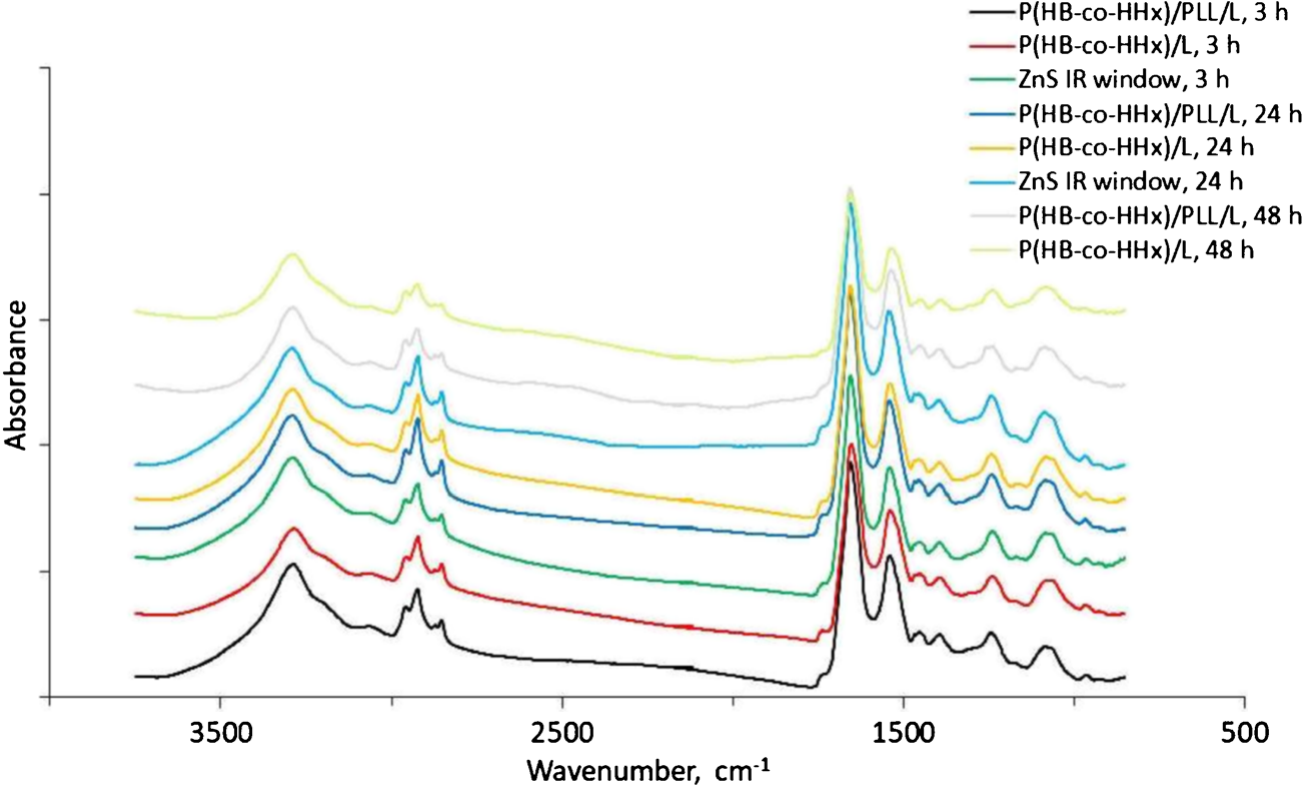
Average IR spectra (3700–850 cm^−1^) from all data sets of imprints and ZnS IR windows with cells after different culture times

IR spectra were collected at specified locations by mapping the imprinted area and, for the cells cultured directly on IR windows for 3 and 24 h, at the positions where cell clusters were clearly observed. Samples of cells on IR windows at 48 h could not be measured due to insufficient material (the spectra acquired showed poor signal-to-noise ratios), as the cells became detached from the window during the processing performed for the measurements, most likely because the high proliferation of cells led to the formation of a detachable sheet due to the relatively low contact surface area compared to the fibers. For each sample, at least 30 spectra were acquired and the entire data set for all samples included over 850 spectra. Figure 3 presents the average spectra obtained for all substrate types and at various culture times in the spectral interval between 3750 and 850 cm^−1^. In order to compare the results obtained for each group, their corresponding second-derivative spectra were calculated after performing smoothing, vector normalization, and PCA over the spectral range 3050–2800 cm^−1^, where bands corresponding mainly to lipids are observed, and the 1800–1400 cm^−1^ region, which mainly includes protein and amide I–II bands. The use of the second-derivative spectrum makes it easier to interpret the results since it has a flat baseline, so the positions of the negative peaks observed can be precisely correlated with the bands and shoulders observed in the absorption spectrum. For small cells, the signal-to-noise ratio advantage obtained with the synchrotron source makes this chemometric approach feasible.

**Fig. 4a–b:**
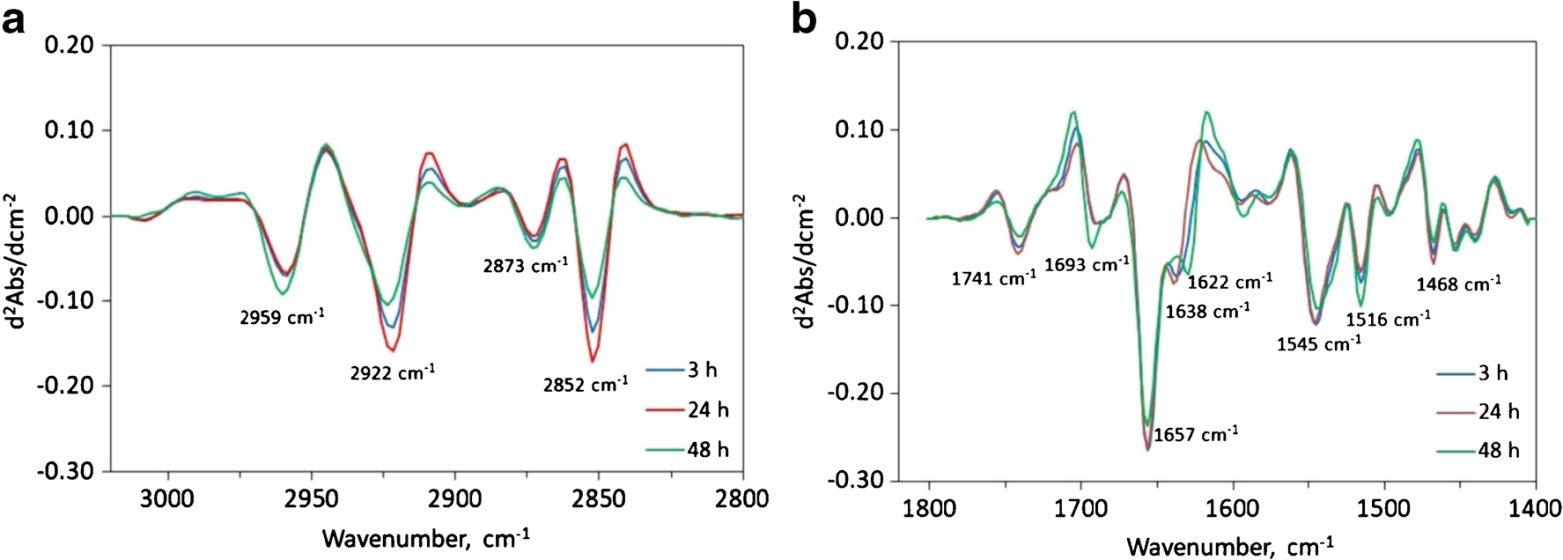
Average second-derivative spectra for NPCs at 3 h (P(HB-*co*-HHx)/PLL/L; P(HB-*co*-HHx)/L, and ZnS IR windows/L), 24 h (P(HB-*co*-HHx)/PLL/L; P(HB-*co*-HHx)/L, and ZnS IR windows/L), and 48 h (P(HB-*co*-HHx)/PLL/L and P(HB-*co*-HHx)/L) after seeding, illustrating the differences between the spectra in the **a** lipid region (3050–2800 cm^−1^) and **b** the amide I–II region (1800–1400 cm^−1^)

In an initial statistical analysis of the spectral data, the culture time was the only experimental variable considered, and a clear division of the spectra into three groups was observed. In Figure 4a, the average second-derivative spectra of NPCs 3, 24, and 48 h after seeding on the three types of substrates are shown. It can be seen that the intensities of the bands associated with the asymmetric and symmetric stretching modes of mainly lipid methylene moieties at 2922 and 2852 cm^−1^, respectively, initially increase but then decrease, such that they have dropped significantly at 48 h. The same pattern of change in relative intensity is also observed for lipid ester carbonyl bands at 1741 cm^−1^ and lipid methylene deformation modes at around 1468 cm^−1^ (Fig. 4b).

The most characteristic bands identified in the second-derivative spectra of NPCs cultured on the P(HB-*co*-HHx) fibers and ZnS IR windows, as well as their assignments, are summarized in Table 1.

**Table 1:** Characteristic FTIR bands observed from neural progenitor cells during adhesion and differentiation on P(HB-*co*-HHx) fibers and ZnS IR windows

Band max., 2nd derivative, cm^−1^	PC-1, 3 h vs 24 h, cm^−1^ (loading)	PC-1, 24 h vs 48 h, cm^−1^ (loading)	Band assignments
2922	2926 (+0.084)	2920 (+0.157)	*ν*_as_ of methylene groups, mainly in lipids
2852	2854 (+0.122)	2852 (+0.188)	*ν*_s_ of methylene groups, mainly in lipids
1741	1743 (+0.026)	1734 (+0.046)	*ν*_s_ of carbonyl ester lipids
1693	1695 (+0.026)	1695 (−0.117)	β-Turn (secondary structure of protein) and antiparallel β-sheets
1657	1660 (+0.196)		α-Helical protein structure
1640	1649 (−0.124)		Unordered protein structures
1638; 1622	1630 (−0.301)	1628 (−0.294)	β-Sheet (secondary structure of protein)
1545	1537 (−0.059)	1535 (−0.070)	Overall protein absorbance
1516	1512 (−0.087)	1516 (−0.094)	C=C stretching in aromatic amino acids and β-turn (secondary structure of protein)

A detailed review of the spectral profiles from cells on each type of substrate (P(HB-*co*-HHx)/PLL/L, P(HB-*co*-HHx)/L, and ZnS/L) at given culture times showed only minor fluctuations in the lipid band intensities (data not shown).

When IR spectra were grouped according to culture time, in the region between 1700 and 1600 cm^−1^ (amide I band, Fig. 4b), a decrease in the intensity of the band attributed to α-helix structure (1657 cm^−1^) was observed at 48 h [42]. A shift of more than 15 cm^−1^ in the band associated with β-sheet structure [42], from 1638 to 1622 cm^−1^, and an increase in the band intensity at 1693 cm^−1^ were also observed; the latter is generally assigned to β-turns and antiparallel β-sheet structures [41]. Also, these changes appear to be correlated with those observed for the amide II [43] peak at 1516 cm^−1^.

An assessment of the amide I band for cells on each type of substrate at the studied culture times showed that the presence of α-helix structures is favored on ZnS substrates, while β-sheet structures are more likely to be formed on P(HB-*co*-HHx)/L fibers (see Fig. S3a and b in the ESM). The FTIR cell profiles on P(HB-*co*-HHx)/PLL/L initially exhibit an intermediate behavior, whereas all cells grown on the polymer fibers show similar spectra at 48 h (see Fig. S3c in the ESM).

### Statistical analysis

**Fig. 5:**
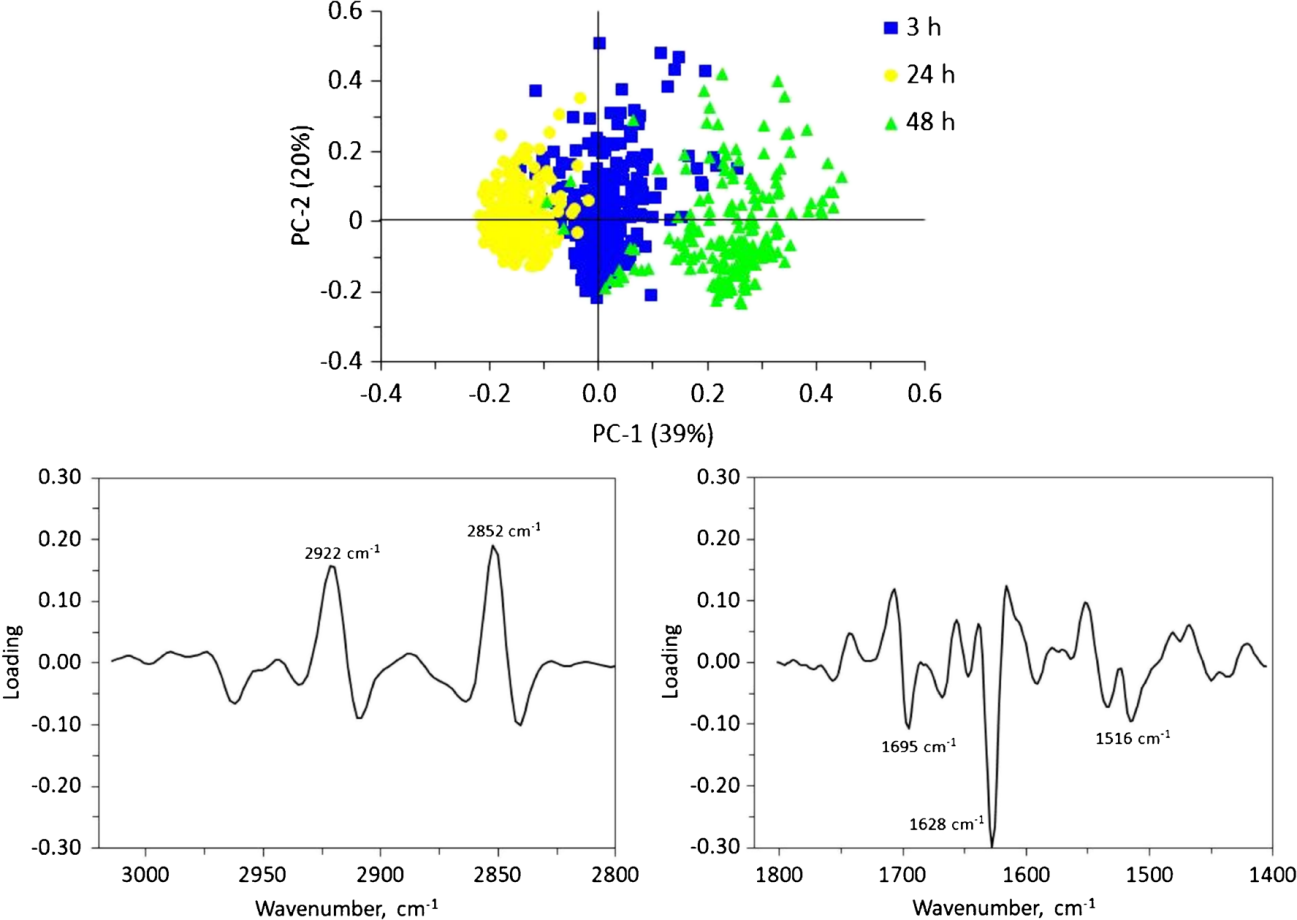
PCA plots for PC-1 vs. PC-2 scores (*top*) and PC-1 loadings (*bottom*) for the spectra of neural progenitor cells on the three types of substrates. The spectral regions included in the data analysis were 3050–2800 cm^−1^ (*bottom left*) and 1800–1400 cm^−1^ (*bottom right*)

Multivariate analysis was implemented to identify possible trends in the changes observed in the entire spectral data set. Principal component analysis (PCA) was performed on the second-derivative spectra calculated from all of the spectra acquired, considering the spectral regions 3050–2800 cm^−1^ and 1800–1400 cm^−1^ in the model. Two-dimensional score plots of PC-1 vs. PC-2 were found to provide adequate clustering of spectra from the cells as a function of cluster time (see the upper part of Fig. 5). Unsupervised cluster analysis of these data yielded classification percentages of 93.7, 97.2 and 91.1% of all samples at 3, 24, and 48 h of culture time, respectively. A closer examination of the corresponding loading plots of PC-1 (Fig. 5, bottom) revealed that the main bands contributing to cluster formation were the methylene lipid bands at 2922 and 2852 cm^−1^, the amide I bands at 1695 and 1628, and the amide II bands at 1545 and 1516 cm^−1^. The outcome of PCA for pairwise comparisons of culture times (3 h vs. 24 h and 24 h vs. 48 h) are illustrated in Fig. S4 (see the ESM). As indicated previously, a reduction in lipid band intensity with increasing culture time was observed. Also, the main changes observed in the amide I–II spectral region are those associated with the increase in band intensities mentioned above and the decrease in the intensity of the band at 1660 cm^−1^ during the initial stages of culture.

**Fig. 6a–c:**
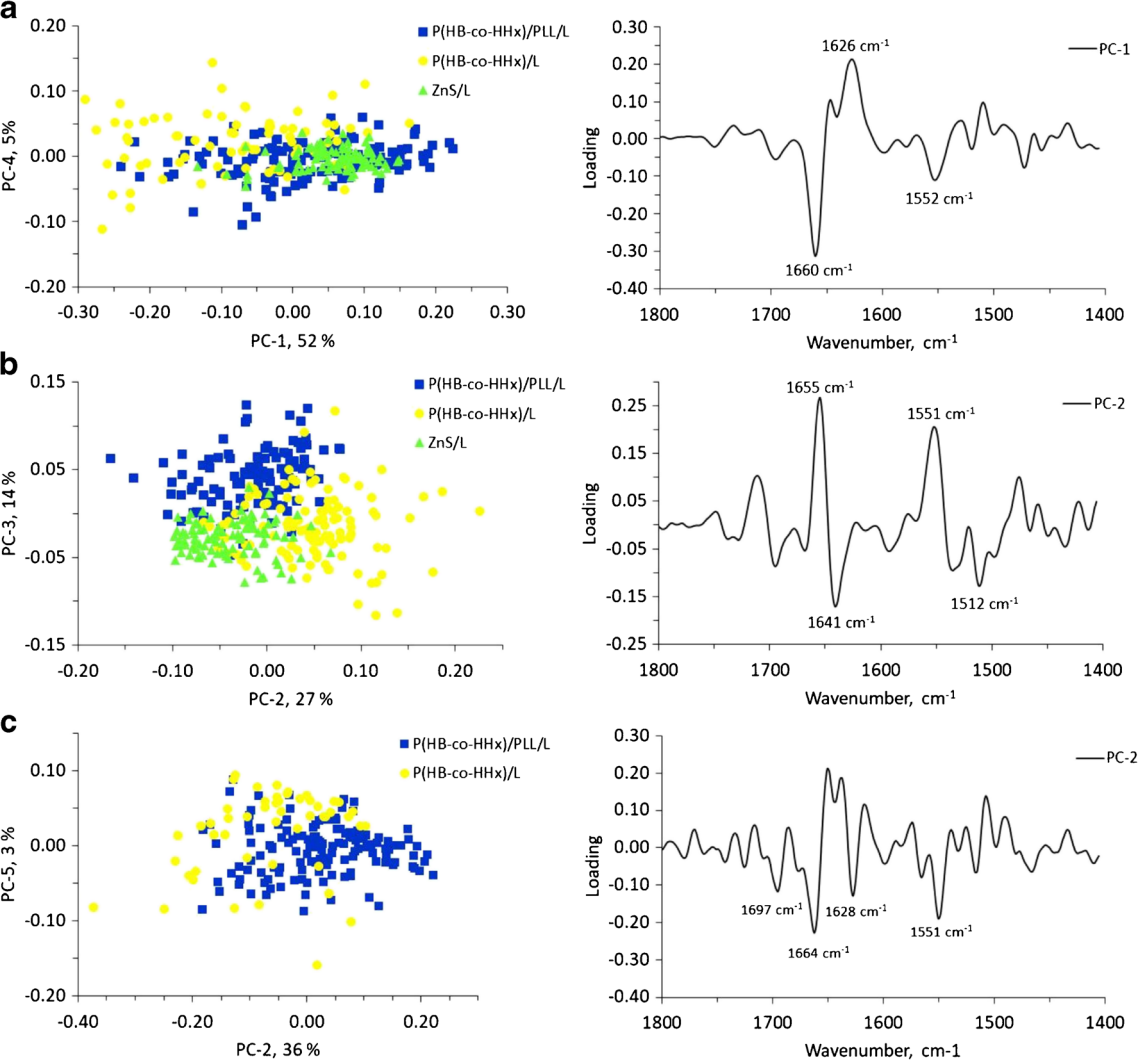
Plots of PCA scores and loadings for the spectra from NPCs at **a** 3 h, **b** 24 h, and **c** 48 h after seeding on the three types of substrates [P(HB-*co*-HHx)/PLL/L (*blue squares*), P(HB-*co*-HHx)/L (*yellow circles*), and ZnS/L (*green triangles*)]. The spectral region included in the analysis was 1800–1400 cm^−1^

A PCA performed on the second-derivative spectra of cells grouped by type of substrate yielded improved clustering of the biochemical profiles as a function of culture time, particularly for the substrates P(HB-*co*-HHx)/PLL/L and ZnS/L (see Fig. S5a and c in the ESM). However, the P(HB-*co*-HHx)/L scaffolds showed a broader overlap (see Fig. S5b in the ESM). It was not possible to distinguish between substrate types and coatings at each culture time by analyzing the spectral regions (lipid and amide I–II bands combined). Since, as indicated previously, all of the substrates showed similar lipid profiles at a given culture time, an analysis that only took into account the spectral region between 1800 and 1400 cm^−1^ was performed. Figure 6 shows selected 2D PCA score plots for the amide I–II spectral region of NPCs at culture times of 3, 24, and 48 h on the three types of substrates studied: P(HB-*co*-HHx)/PLL/L (PC-1 vs PC-4), P(HB-*co*-HHx)/L (PC-2 vs PC-3), and IR windows/L (PC-2 vs PC-5). Although a significant overlap between the groups is apparent, particularly at 3 h, there is a noticeable tendency to segregate according to the type of substrate/coating. The loading plots in Fig. 6 show that these results are mainly due to changes in the previously mentioned bands of the NPC profiles: the amide I bands associated with β-turns (~1690 cm^−1^), α-helices (~1660 cm^−1^), and β-sheets (1638–1622 cm^−1^) of proteins, as well as the amide II band at 1512 cm^−1^, which is closely related to β-turns [43].

## Discussion

The FTIR spectrum of NPCs on P(HB-*co*-HHx) fibers showed a marked decrease in lipid band intensity at the longest culture time studied, 48 h. This observation is consistent with previous observations which showed a reduction in lipid content that was associated with a loss of stem cell pluri- or multipotency [29]. However, there is still no clear consensus about this, as the opposite behavior—an increase in lipid production with cell differentiation—has also been reported [31]. Several outcomes may be anticipated, depending on the differentiation path of the NPCs. NPC differentiation to an oligodendrocyte-like cell could increase the intensity of the lipid bands due to an increase in myelin. On the other hand, if NPCs differentiate to neuron or astrocyte-like cells, a reduction in the lipid signal intensity might be observed. In our case, previous results have shown that on standard culture plates with the culture medium used in this study, neurospheres differentiate to an intermediate stage, maintaining their differentiation potential to some extent [36].

The changes observed in the amide I band, corresponding to an increase in the amount of β-sheets and β-turns present, support the above statement. The immunochemistry results show that the GFAP signal increased progressively with increasing culture time, particularly on ZnS/L IR windows and P(HB-*co*-HHx)/PLL/L fibers. The presence of the protein nestin, a biomarker for NPCs, was more prominent during the initial stages of cell culture.

An increase in the heterogeneity of the biochemical profiles of cells with increasing culture time can be anticipated because, for the electrospun polyester fiber substrates, the likelihood of finding cells at different stages of differentiation would increase over time. This would lead to a broadening of the spectral cluster determined by PCA. Although the outcome of this report is influenced by the limited set of variables studied and the duration of cell culture, it is worth pointing out that the cells on fibers coated only with laminin exhibited the greatest overlap between the three distinct populations identified by FTIR microspectroscopy. On the other hand, the NPCs on ZnS IR/L windows showed the narrowest distributions. Thus, although the culture time is a major influence on the cell IR biochemical profile, these observations suggest that the type of substrate/coating present affects the cell differentiation pathway, and could be adjusted to modulate the cellular response appropriately. In this regard, given the cell profile heterogeneity and the short exposure times to the biomaterials, the observation that spectral clustering was influenced by the type and coating of the cell scaffolds used is remarkable. These findings are in agreement with results from other studies showing that the surface chemistry and topography substantially influence the outcome of NPC differentiation [19], and highlight the potential of synchrotron FTIR microspectroscopy for studying the interactions between cells and biomaterials. Nevertheless, further experiments incorporating different surface chemistries and extended culture times are required to attain a more robust response.

## Conclusions

Neural progenitor cells were cultured on electrospun P(HB-*co*-HHx) fiber substrates coated with either laminin or poly-L-lysine/laminin. At different times after cell seeding, the samples were fixed and the cellular material on the fibers was partly transferred onto IR windows using a modified touch imprint cytology method. This strategy proved to be very effective and enabled the biochemical profiles of the NPCs that evolved on the coated polymer fibers to be determined with SIRMS. The intensities of bands in the lipid and amide I–II regions were observed to be influenced by the substrate type and coating and the culture time. These results demonstrate the important insights that SIRMS studies can provide into the responses of NPCs to biomaterials (in this case to the morphology and surface chemistry of polymeric scaffolds), suggesting that such studies could be a fundamental tool in the preparation and optimization of cellular scaffolds for CNS tissue engineering and regenerative medicine.

## Electronic supplementary material

ESM 1 (PDF 702 kb)
