# Supplementary material for: Biochemical profiling of rat embryonic stem cells grown on electrospun polyester fibers using synchrotron infrared microspectroscopy
Source: Anal Bioanal Chem. 2018 Apr 18;410(16):3649–60. doi: 10.1007/s00216-018-1049-z (PMC5956007; doi:10.1007/s00216-018-1049-z)
Supplement: Supplementary file 1 — (PDF 702 kb) [file 216_2018_1049_MOESM1_ESM.pdf]

Analytical and Bioanalytical Chemistry

Electronic Supplementary Material

Biochemical profiling of rat embryonic stem cells grown on electrospun polyester fibers using synchrotron infrared microspectroscopy

Ernesto Doncel-Pérez, Gary Ellis, Christophe Sandt, Peter S. Shuttleworth, Agatha Bastida, Julia Revuelta, Eduardo García-Junceda, Alfonso Fernández-Mayoralas, Leoncio Garrido

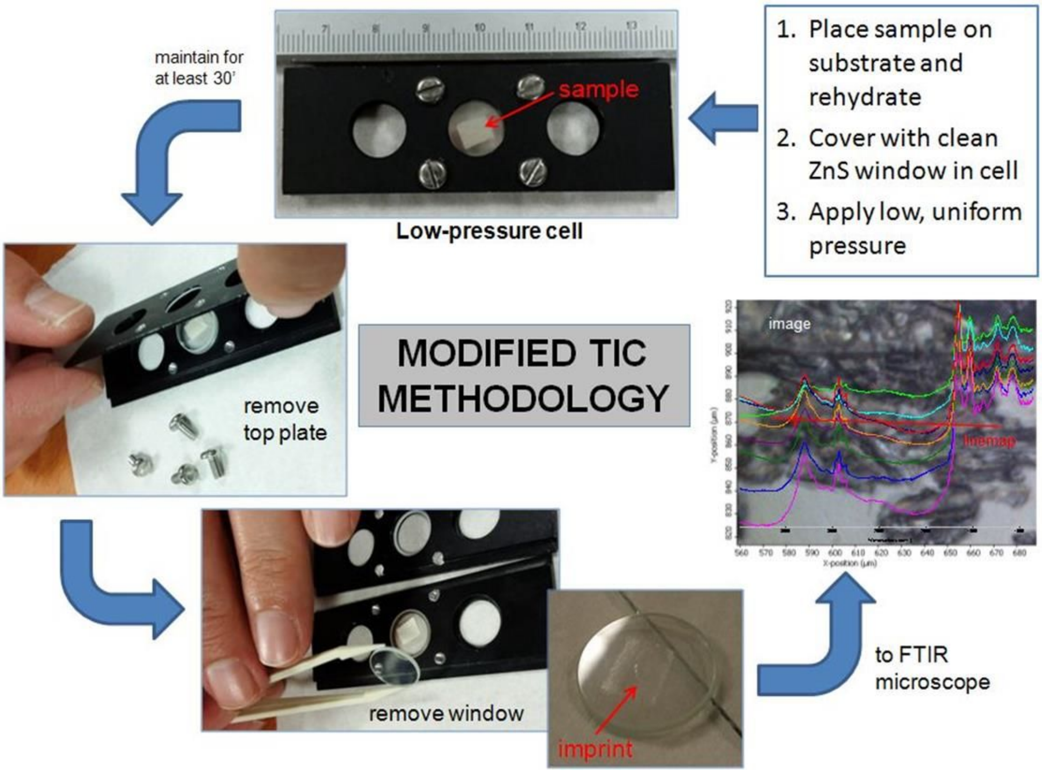

Fig. S1 Illustration of the modified "touch imprint cytology" (TIC) method used to partially transfer some of the biological material on the P(HB-*co*-HHx) scaffolds to the IR transparent window

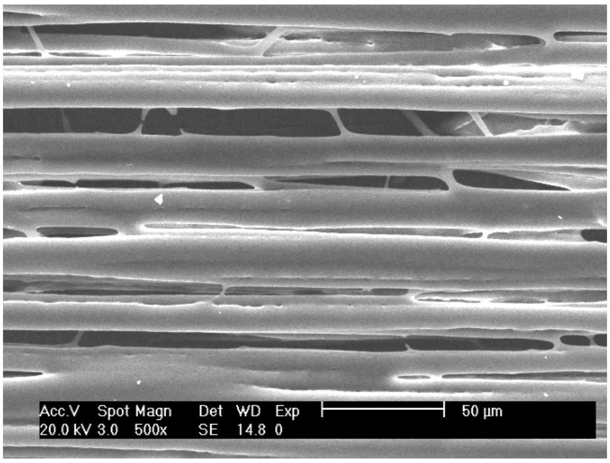

Fig. S2 Representative SEM image corresponding to fibers of P(HB-*co*-HHx) prepared via electrospinning of a solution of the copolymer in a mixture of dichloromethane and 1,1,1,3,3,3-hexafluoro-2-propanol using the experimental conditions described in Methods

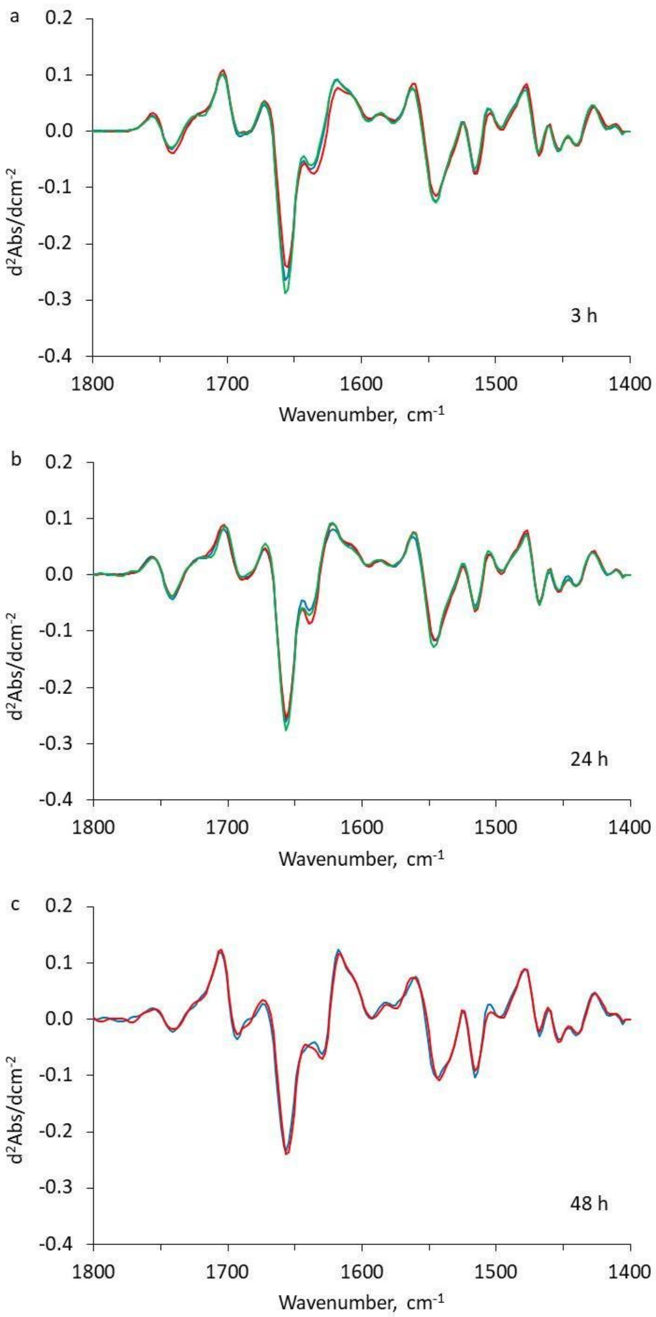

Fig. S3 Average second derivative spectra corresponding to NPCs on (green) ZnS IR windows/L; (red) P(HB-*co*-HHx)/L, and (blue) P(HB-*co*-HHx)/PLL/L fibers at 3, 24 and 48 h after seeding, illustrating the differences in the amide I and II (1800-1400 cm^{-1}) region

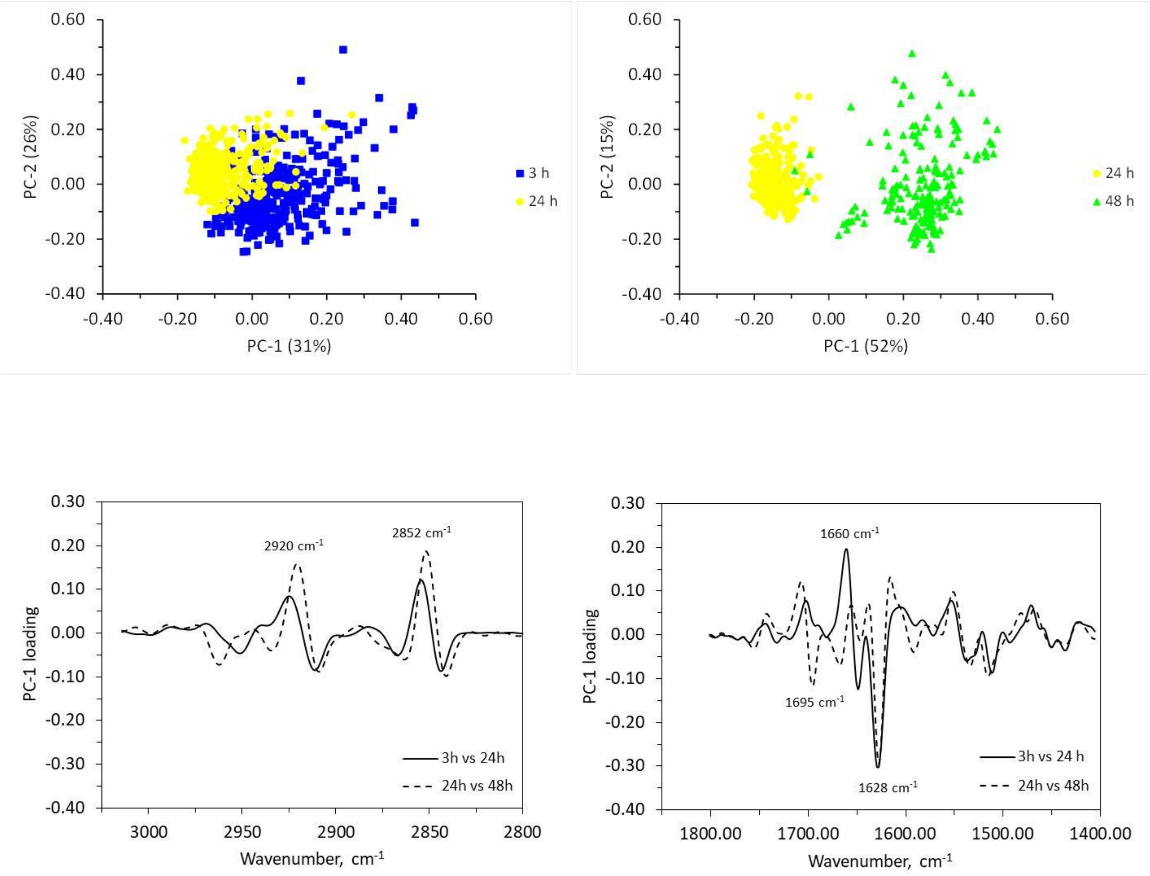

Fig. S4 Plots of PCA (top) PC-1 *vs*. PC-2 scores and (bottom) PC-1 loadings for the spectra of neural progenitor cells on the three types of substrates, illustrating the spectral differences between the cell cultures at 3 h *vs*. 24 h and 24 h *vs*. 48 h. The spectral regions included in data analysis were 3050-2800 cm^{-1} and 1800-1400 cm^{-1}

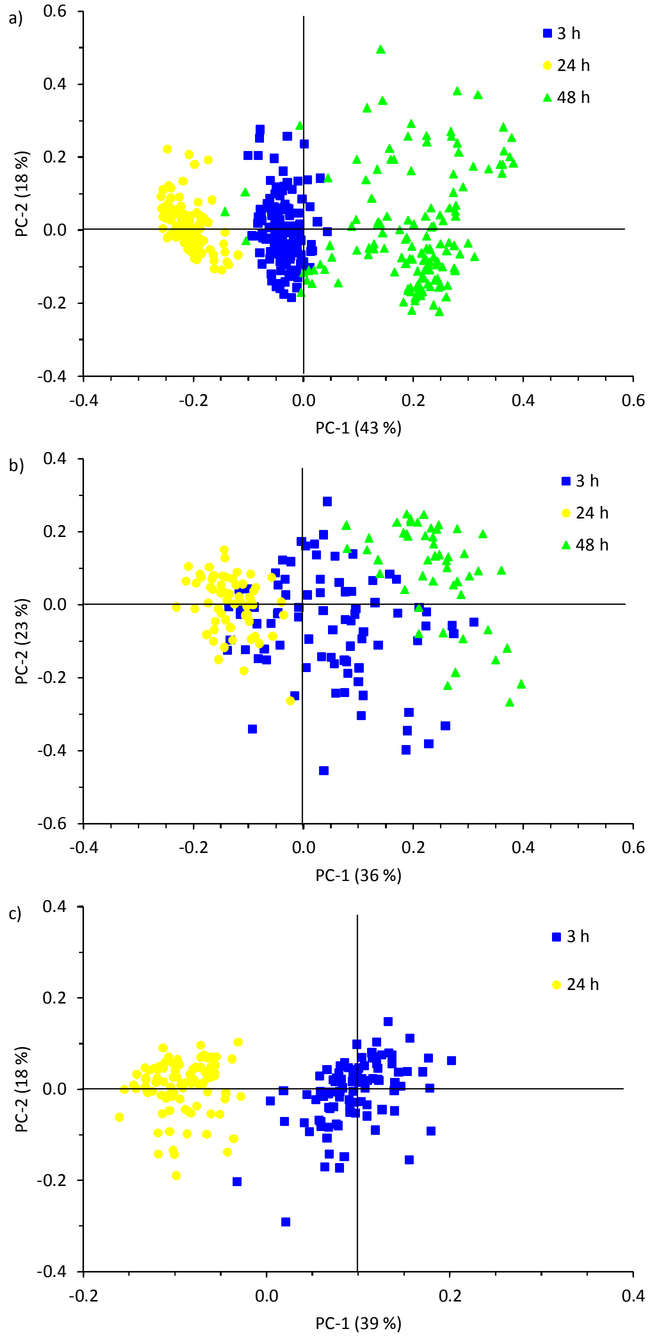

Fig. S5 Plots of PCA scores for the spectra of NPCs on (a) P(HB-co-HHx)/PLL/L; (b) P(HB-co-HHx)/L, and (c) ZnS IR window/L, at 3 h (blue, squares), 24 h (yellow, circles) and 48 h (green, triangles). The spectral regions included in data analysis were 3050-2800 cm^{-1} and 1800-1400 cm^{-1}
